# Association of uric acid and uric acid to creatinine ratio with chronic kidney disease in hypertensive patients

**DOI:** 10.1186/s12882-021-02521-9

**Published:** 2021-09-16

**Authors:** Nathalia Rabello Silva, Camila Evangelista Torres Gonçalves, Danilo Lemes Naves Gonçalves, Rosângela Minardi Mitre Cotta, Luciana Saraiva da Silva

**Affiliations:** 1 School of Medicine, Federal University of Uberlândia, Minas Gerais, 1720, Pará Avenue, Block 2U, Campus Umuarama, Code postal: 38400-902 Uberlândia, Brazil; 2 Department of Nutrition and Health, Federal University of Viçosa, Minas Gerais Viçosa, Brazil

**Keywords:** Chronic kidney disease, Uric acid, Creatinine, Hypertension

## Abstract

**Background:**

Recent studies have shown that the serum uric acid/creatinine ratio (SUA/SCr) is a better predictor of chronic kidney disease (CKD) than serum uric acid (SUA) isolated. The aim of the present study was to evaluate the association of isolated SUA and the SUA/SCr with CKD in hypertensive patients.

**Methods:**

Cross-sectional study conducted with hypertensive patients followed-up by the Primary Health Care Service (PHC). Sociodemographic, economic, lifestyle, clinical, anthropometric, and biochemical variables were evaluated. The association between SUA parameters (quartiles of SUA and quartiles of SUA/SCr) and CKD was evaluated by bivariate and multivariate logistic regression. The association between SUA parameters (SUA and SUA/SCr) and estimated glomerular filtration rate (eGFR) was evaluated by linear regression. The analyses were performed considering four adjustment models. SUA and SUA/SCr were compared by receiver operating characteristic (ROC) curve.

**Results:**

In the fully adjusted model, SUA was positively associated with the presence of CKD (OR = 6.72 [95 % CI 1.96–22.96]) and inversely associated with eGFR (β Coef. = -2.41 [95 % CI -3.44; -1.39]). SUA/SCr was positively associated with eGFR (β Coef. = 2.39 [1.42; 3.36]). According to the ROC curve, the SUA is a better predictor of CKD than the SUA/SCr.

**Conclusions:**

Elevated levels of isolated SUA were associated with CKD and eGFR. However, the SUA/SCr was not associated with CKD. We do not recommend using the SUA/SCr to predict CKD in hypertensive patients.

## Introduction

Chronic Kidney Disease (CKD) is considered a growing public health problem worldwide, reaching about 850 million people [1]. According to the Global Burden of Disease Study, in 2017, CKD accounted for 1.2 million deaths [2].

In the past decades, the function of serum uric acid (SUA) in the genesis and evolution of CKD has motivated numerous studies. Recent studies have shown that soluble SUA exhibits a behavioral duality acting as pro-oxidant within the cell and antioxidant in the extracellular environment [3, 4]. The remnant of circulating SUA accounts for more than half of the antioxidant potential of human blood [5]. However, when it is inside the cells, it exhibits a pro-oxidant behavior [6]. A meta-analysis conducted in 2014 with 190,718 participants found a significant positive association between high levels of SUA and incidence of CKD [7], while other studies found no association [8–10], indicating controversies about the role of SUA in CKD.

SUA results from purine metabolism and is excreted mainly by the kidneys. In patients with CKD, the level of SUA may be increased due to decreased excretion capacity of the kidneys [7]. On the other hand, some studies suggest that hyperuricemia causes renal injury by vasoconstriction mediated by endothelium dysfunction, activation of the renin-angiotensin system and epithelial changes in renal tubular cells [5, 11]. SUA may also be associated with the development of CKD through some factors, such as organ toxicity and worsening of risk factors for CKD, such as arterial hypertension (AH) [5, 12].

Recent studies [9, 13–15] have shown that serum uric acid to creatinine ratio (SUA/SCr) is a better predictor of CKD incidence than isolated SUA in patients with type 2 diabetes mellitus (DM), besides being considered a good biomarker for detecting the pathogenesis of metabolic syndrome [15–18]. Thus, the SUA/SCr can provide new information to explain the association between SUA and CKD, however, studies are still scarce and clinical data of this indicator are limited [15]. Furthermore, to the best of our knowledge, the relationship between the SUA/SCr and CKD has not yet been evaluated in the population with AH. Therefore, the aim of this study was to evaluate the association of isolated SUA and the SUA/SCr with CKD in patients with AH.

## Methods

 This is a cross-sectional study conducted with patients with AH followed-up by the Primary Health Care Service (PHC) in the municipality of Porto Firme, Minas Gerais, Brazil.

The sample was defined considering the total number of registered hypertensive patients (n = 697), prevalence of 50 % of the studied phenomenon, 5 % of margin of sampling error and confidence level of 95 %. The sample calculation resulted in a minimum sample of 248 individuals. The sample calculation was performed using the Statcalc program of Epi-Info® version 7.2.

The inclusion criteria were: individuals aged 18 years or older, with AH and who agreed to participate in the study after proper clarification. Exclusion criteria were: individuals who presented severe clinical conditions that required specialized care, as well as pregnant women and individuals with a history of alcohol and/or drug abuse. For the selection of participants, we used a convenience sample. All hypertensive patients followed by PHC were invited to participate in the study and the final sample was composed of 293 individuals.

At the beginning of the study, all study participants had a previous diagnosis of AH and were taking antihypertensive medications. According to the Brazilian Guideline of Arterial Hypertension [19], the definition of AH consists of persistent elevation of blood pressure (BP), that is, systolic BP (SBP) greater than or equal to 140 mmHg and/or diastolic BP (DBP) greater or equal to 90 mmHg, measured with the correct technique, at least two different occasions, in the absence of antihypertensive medication.

Data were collected through individual interviews, anthropometric and biochemical evaluations. A semi-structured interview guide was used as an instrument to collect information, addressing sociodemographic, economic, lifestyle and clinical variables. For the evaluation of physical activity, the short version of the International Physical Activity Questionnaire proposed by the World Health Organization (WHO) and validated in Brazil was used [20].

The anthropometric measurements evaluated were weight, height and waist circumference (WC). The weight was obtained using an electronic scale, with a capacity of 150 kg and division of 50 g; and height was measured using a portable anthropometer, consisting of a metal platform for positioning individuals and a demountable wooden column containing millimeter tape and cursor for reading, according to the techniques proposed by Jelliffe [21]. Body mass index (BMI) was calculated by the relationship between weight and squared height and classified according to the WHO criteria [22] for adults, and Lipschitz [23] for the elderly. WC was measured in centimeters using an inextensible tape at the midpoint between the iliac crest and the outer face of the last rib. The values were classified in relation to the risk for cardiovascular diseases and metabolic complications according to the cutoff points proposed by the WHO [22].

For renal function analysis, serum creatinine, SUA and albuminuria (by 24-hour urine collection) were evaluated. CKD was identified using the estimation of glomerular filtration rate (eGFR) using the CKD-EPI formula, currently recommended by KDIGO [24]. Once the presence of CKD was identified (eGFR < 60mL/min/1.73 m² and/or albuminuria > 30 mg/g), creatinine and albuminuria tests were repeated after three months to confirm the diagnosis, as recommended by KDIGO [24]. The SUA/SCr was calculated by dividing the serum values of uric acid by creatinine.

 Participants personally received information on the 24-hour urine collection procedure, written instructions and recipients for collection, and were instructed to maintain a usual diet during the day and fast 12 h before collection. On the scheduled day, the participants attended the accredited laboratory for the delivery of urine and blood collection. Urine volume below 500 mL in 24 h was not included in the analysis. Biological material collection and analysis were performed in a single laboratory, using commercial kits.

For quantitative data analysis, the Software SPSS Statistics for Windows (Version 20.0) was used. The descriptive analysis of the study participants was presented according the presence/absence of CKD. SUA parameters were presented by sex. The association between CKD and SUA parameters (quartiles of SUA and quartiles of SUA/SCr) was evaluated by bivariate and multivariate logistic regression. The association between the continuous values of the eGFR and the SUA parameters (SUA and SUA/SCr) was evaluated by linear regression. The analyses were performed considering four models:

Model 1: adjusted for sex and age;

Model 2: adjusted for Model 1 + schooling, marital status and income;

Model 3: adjusted for Model 2 + tobacco, alcohol, diabetes, BMI, WC, time of AH and physical activity;

Model 4: adjusted for Model 3 + glucose, total cholesterol, HDL cholesterol, LDL cholesterol, triglycerides, urea and blood pressure controlled.

A comparison of SUA and SUA/SCr in CKD were analyzed in terms of a receiver operating characteristic (ROC) curve. A ROC curve is a plot between sensitivity (Y-axis) versus false positive (X-axis), obtained for different cutoff points. Areas under the curve (AUC) of the ROC curves and their 95 per cent confidence intervals (CI) were evaluated as a measure of diagnostic accuracy. Greater AUC of the ROC curve indicated better markers of the study. The AUC values were classified as: excellent (0.90–1.00), good (0.80–0.90), regular (0.70–0.80); poor (0.60–0.70), bad (0.50–0.60) and insufficient as a diagnostic test (< 0.50) [25].

## Results

Table 1 shows the sociodemographic, economic, lifestyle, clinical, anthropometric and biochemical characteristics, according to the presence/absence of CKD.

**Table 1:** Characterization of the population according to the presence/absence of CKD

Variables			CKD		p
			No	Yes	
			n (%) or mean (sd)		
Gender		Female	132 (60.8)	85 (39.2)	0.720
		Male	48 (63.2)	28 (36.8)	
Age (years)			62 (12)	72 (9)	< 0.001*
Education		High school or more	15 (65.2)	8 (34.8)	0.077
		Up to 8th grade	14 (58.3)	10 (41.7)	
		Up to 4th grade	114 (66.7)	57 (33.3)	
		Illiterate	37 (49.3)	38 (50.7)	
Civil status		With a partner	117 (64.6)	64 (35.4)	0.152
		No partner	63 (56.2)	49 (43.8)	
Tobacco		Never smoked	113 (59.8)	76 (40.2)	0.626
		Ex-smoker	51 (63.0)	30 (37.0)	
		Smoker	16 (69.6)	7 (30.4)	
Alcohol intake		No	151 (58.8)	106 (41.2)	0.012*
		Yes	29 (80.6)	7 (19.4)	
Physical Activity		Active	126 (65.6)	66 (34.4)	0.042*
		Not active	54 (53.5)	47 (46.5)	
Diabetes Mellitus		No	144 (61.0)	92 (39.0)	0.766
		Yes	36 (63.2)	21 (36.8)	
Time with AH		< 10 years	114 (65.5)	60 (34.5)	0.082
		> 10 years	66 (55.5)	53 (44.5)	
Blood pressure controlled		No	27 (15.0)	17 (15.0)	0.949
		Yes	153 (85.0)	96 (85.0)	
Antihypertensive drugs		Thiazide diuretics	101 (56.1)	63 (55.8)	0.952
		Angiotensin-converting enzyme inhibitors	56 (31.1)	41 (36.3)	0.366
		Angiotensin receptor blockers	63 (35.0)	41 (36.3)	0.978
		Loop diuretics	19 (10.6)	25 (22.1)	0.012*
		Beta-blockers	37 (20.6)	19 (16.8)	0.422
Overweight		No	51 (52.6)	46 (47.4)	0.028*
		Yes	129 (65.8)	67 (34.2)	
Glucose (mg/dL)			104.11 (36.97)	107.05 (32.49)	0.487
Total cholesterol (mg/dL)			202.52 (37.85)	201.34 (36.00)	0.790
HDL cholesterol (mg/dL)			47.72 (8.52)	48.54 (8.98)	0.431
LDL cholesterol (mg/dL)			122.70 (36.98)	120.69 (35.84)	0.648
Tryglicerides (mg/dL)			149.82 (76.25)	151.73 (97.11)	0.851
Urea (mg/dL)			34.8 (5.3)	43.4 (9.2)	< 0.001*
Albuminuria (mg/g)		< 30	145 (64.2)	81 (35.8)	0.204
		30 - 300	31 (51.7)	29 (48.3)	
		> 300	4 (57.1)	3 (42.9)	
Creatinine (mg/dL)			0.93 (0.11)	1.19 (0.27)	< 0.001*
eGFR (mL/min/1.73m²)			72.38 (9.96)	50.72 (8.02)	< 0.001*
**SUA parameters**					
SUA (mg/dL)	Male Female Total		6.24 (1.00) 4.60 (1.05) 5.04 (1.26)	6.74 (1.00) 5.45 (1.24) 5.77 (1.31)	0.038* < 0.001* < 0.001*
SUA/SCr	Male Female Total		5.96 (0.93) 5.22 (1.19) 5.41 (1.17)	4.97 (0.96) 4.90 (1.13) 4.92 (1.09)	< 0.001* 0.053 < 0.001*
Quartiles SUA	Q1 (2.60 – 4.30)	Male Female Total	3 (75.0) 56 (78.9) 59 (78.7)	1 (25.0) 15 (21.1) 16 (21.3)	< 0.001*
	Q2 (4.31 – 5.30)	Male Female Total	6 (85.7) 43 (57.3) 49 (59.8)	1 (14.3) 32 (42.7) 33 (40.2)	
	Q3 (5.31 – 6.30)	Male Female Total	16 (80.0) 27 (56.2) 43 (63.2)	4 (20.0) 21 (43.8) 25 (36.8)	
	Q4 (6.31 – 9.30)	Male Female Total	23 (51.1) 6 (26.1) 29 (42.6)	22 (48.9) 17 (73.9) 39 (57.4)	
Quartiles SUA/SCr	Q1 (1.72 – 4.48)	Male Female Total	4 (40.0) 35 (54.7) 39 (52.7)	6 (60.0) 29 (45.3) 35 (47.3)	0.005*
	Q2 (4.49 – 5.15)	Male Female Total	5 (33.3) 33 (55.9) 38 (51.4)	10 (66.7) 26 (44.1) 36 (48.6)	
	Q3 (5.16 – 6.06)	Male Female Total	15 (60.0) 33 (68.8) 48 (65.8)	10 (40.0) 15 (31.2) 25 (34.2)	
	Q4 (6.07 – 11.49)	Male Female Total	24 (92.3) 31 (67.4) 55 (76.4)	2 (7.7) 15 (32.6) 17 (23.6)	

The prevalence of CKD was 38.6 % (n = 113). Individuals with CKD had a higher mean age. Individuals without CKD had a higher prevalence of alcohol consumption, physical activity and overweight. Regarding renal function parameters, there was a significant difference for urea, creatinine, eGFR, SUA and SUA/SCr. SUA values ​​were higher in CKD patients and in men than in women. SUA/SCr values ​​were lower in CKD patients and similar between men and women.

Concerning regression analyses (Table 2), SUA was positively associated with the presence of CKD (OR = 6.72 [95 % CI 1.96–22.96]) and inversely associated with eGFR (β Coef. = -2.41 [95 % CI -3.44; -1.39]) in the adjusted model. Regarding the SUA/SCr, we found a positive association with eGFR (β Coef. = 2.39 [1.42; 3.36]) in model 4.

**Table.2:** Association of SUA parameters (SUA and SUA/SCr) with CKD and eTFG

	Unadjusted model	Model 1	Model 2	Model 3	Model 4
**SUA x CKD**					
Q1 (2.60–4.30)	1	1	1	1	1
Q2 (4.31–5.30)	2.48 (1.22–5.03)	3.32 (1.48–7.43)	3.27 (1.41–7.57)	3.16 (1.32–7.55)	1.86 (0.71–4.86)
Q3 (5.31–6.30)	2.14 (1.02–4.49)	4.41 (1.83–10.62)	4.66 (1.88–11.55)	4.55 (1.76–11.72)	2.80 (0.99–7.91)
Q4 (6.31–9.30)	4.95 (2.38–10.31)	16.37 (5.81–46.08)	17.90 (6.12–52.37)	18.71 (6.14–57.01)	6.72 (1.96–22.96)
**SUA x eGFR**					
Coefficient β (CI 95 %)	-3.02 (-4.19 – -1.86)	-3.73 (-4.77 – -2.69)	-3.69 (-4.76 – -2.62)	-3.68 (-4.78 – -2.58)	-2.41 (-3.44 – -1.39)
**SUA/SCr x CKD**					
Q1 (1.72–4.48)	1	1	1	1	1
Q2 (4.49–5.15)	1.05 (0.55–2.01)	0.99 (0.48–1.05)	0.94 (0.44–2.01)	0.96 (0.44–2.12)	0.93 (0.36–2.40)
Q3 (5.16–6.06)	0.58 (0.29–1.12)	0.85 (0.40–1.82)	0.89 (0.41–1.93)	0.78 (0.34–1.75)	0.62 (0.23–1.67)
Q4 (6.07–11.49)	0.34 (0.16–0.70)	0.38 (0.17–0.84)	0.38 (0.17–0.85)	0.37 (0.15–0.86)	0.38 (0.14–1.04)
**SUA/SCr x eGFR**					
Coefficient β (CI 95 %)	3.74 (2.41–5.07)	3.07 (1.99–4.15)	3.12 (2.02–4.22)	3.29 (2.18–4.41)	2.39 (1.42–3.36)

Model 1: adjusted for sex and age;

Model 2: adjusted for Model 1 + schooling, marital status and income;

Model 3: adjusted for Model 2 + tobacco, alcohol, diabetes, BMI, WC, time of AH and physical activity;

Model 4: adjusted for Model 3 + glucose, total cholesterol, HDL cholesterol, LDL cholesterol, triglycerides, urea and blood pressure controlled

**Fig. 1:**
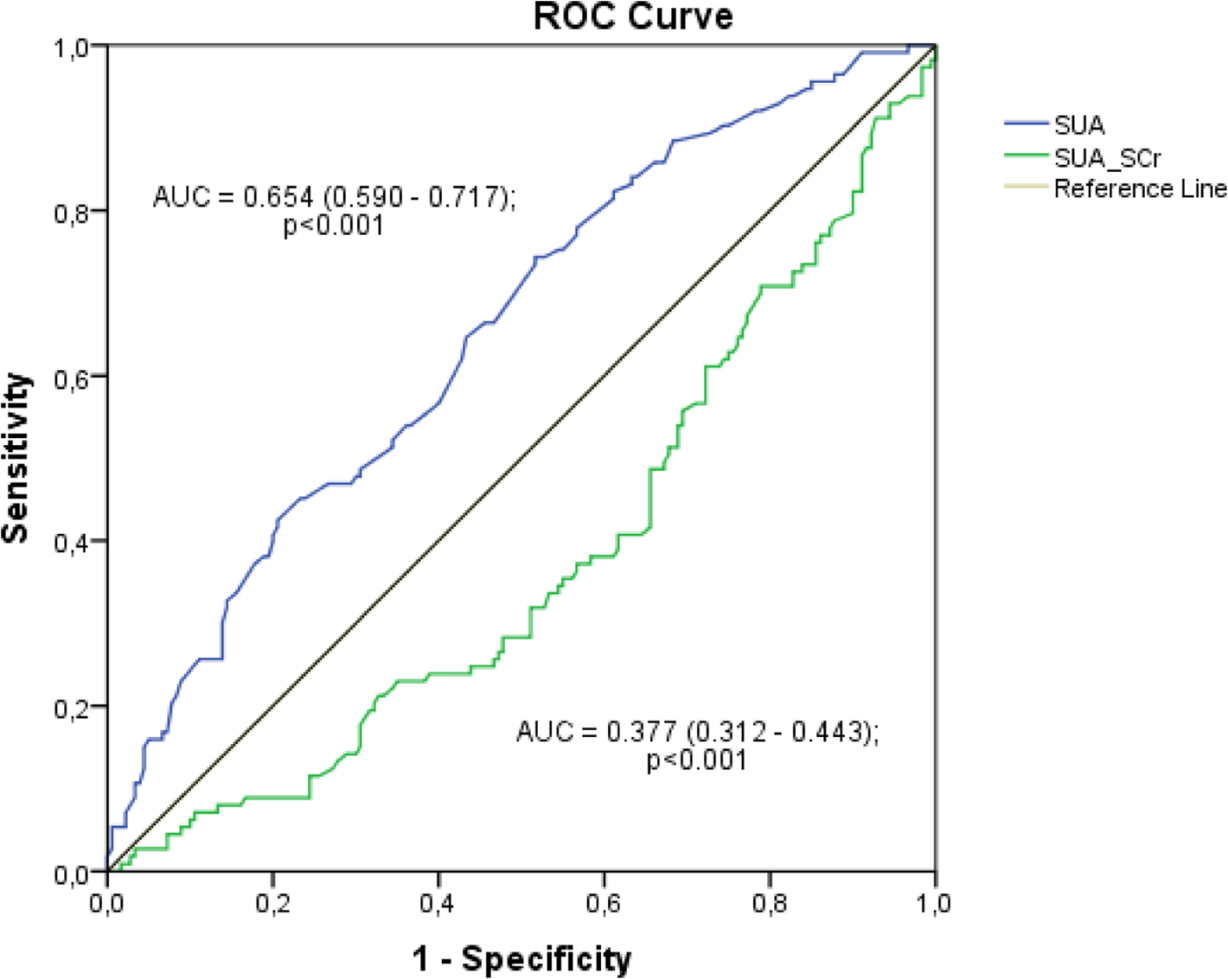
ROC curve of SUA and SUA/Scr as predictors of CKD

Figure 1 shows the ROC curve for SUA and SUA/SCr as predictors of CKD. SUA had a greater AUC than SUA/SCr, hence from the curve, although it is a poor predictor, SUA can be considered better than SUA/SCr.

## Discussion

The findings of this study showed a positive and independent association of SUA with CKD (OR = 6.72; CI 95 % 1.96–22.96), an inverse and independent association of SUA with eGFR (β Coef. = -2.41; CI 95 % -3.44 – -1.39) and a positive and independent association of the SUA/SCr with the eGFR (2.39; IC 95 % 1.42–3.36). Thus, the high levels of isolated SUA seem to be related to CKD and reduced eGFR, which is in line with other studies [7, 12, 26, 27].

The relationship between SUA and CKD also was found in many longitudinal studies. A cohort study with 13,133 health adults found an increased risk of new-onset CKD with the elevated SUA level [28]. The 26,971 individuals evaluated in the Uric Acid Right for Heart Health (URRAH) Project (which more than a half were hypertensive patients) presented 10 times more hyperuricemia when the kidney function was mildly decreased compared to the normal eGFR (> 90 ml/min per 1.73m^2^) [29].

Some potential mechanisms may explain the relationship between SUA and CKD. SUA leads to the oxidative stress and endothelial dysfunction with activation of the renin-angiotensin-aldosterone system [30–32], besides inducing the activation of inflammatory pathways [33]. Such pathophysiological mechanisms may justify the role of SUA in the incidence of CKD. Moreover, SUA is eliminated mainly in urine, thus being reasonable that the level of SUA increases in reduced eGFR and CKD due to impaired clearance of SUA [32].

As the renal clearance of SUA is affected by renal function, the SUA/SCr (also known as the normalized renal function SUA) was created to minimize this interference. In our study, despite the association of SUA with CKD, when we analyzed the SUA/SCr, there was no association with CKD. On the other hand, in agreement with the study of Ephraim et al. [34], there was a positive association with eGFR, and the increase of 1 unit in the index SUA/SCr increased the eGFR values by 2.39 mL/min/1.73 m².

Regarding the SUA/SCr, to the best of our knowledge, this is the first study conducted specifically with hypertensive patients. In other populations, the results remain controversial. A study conducted in Thailand with 446 diabetic patients aimed to verify whether this index SUA/SCr could be used as a biomarker of eGFR and CKD, and the outcome found was favorable to the use of the index [13]. Another cohort study conducted in Japan with 344 diabetic patients aimed to demonstrate whether the SUA/SCr is a useful predictor to indicate decreased renal function, and the results of this publication showed that the use of this index was independently associated with the decline in renal function of the sample studied; however, the authors highlighted that the mechanism that explains this relationship is still unknown [14]. On the other hand, a recent cross sectional study showed an opposite finding, in 155 diabetic patients the SUA was more accurate to assess the renal dysfunction than the SUA/SCr [34], which corroborates the present study.

Finally, from the analysis of the ROC curve, we do not recommend the use of SUA/SCr to predict CKD in hypertensive patients. Furthermore, our findings support the potential relevance of SUA as a biomarker of CKD. Nevertheless, longitudinal and intervention studies must be conducted to determine whether SUA/SCr can, in fact, contribute to the management of CKD.

In addition, considering that all participants in our study are hypertensive, we highlight the possible role of AH influencing the association between high levels of SUA and CKD. The correlation between SUA and AH is well documented and many studies have reported linear and dose-dependent associations [35–39]. In a study conducted with normotensive adults, there was no association between high SUA and incidence of CKD [40], while another study found that the association between high SUA and CKD was stronger in hypertensive patients than in normotensive adults [41].

In relation to the limitations, our study is cross-sectional, not allowing inferring causality from the results, but enabling the formulation of hypotheses that should be confirmed with future studies. In addition, we had no information related to the presence of gout and the use of hypouricemiants, which may be important confounding factors to be included in the analyses.

## Data Availability

The datasets used and analyzed during the current study are available from the corresponding author on reasonable request.

## References

[1] Jager KJ, Kovesdy C, Langham R, Rosenberg M, Jha V, Zoccali C (2019). A single number for advocacy and communication-worldwide more than 850 million individuals have kidney diseases. Nephrology Dialysis Transplantation.

[2] Bikbov B, Purcell CA, Levey AS, Smith M, Abdoli A, Abebe M (2020). Global, regional, and national burden of chronic kidney disease, 1990–2017: a systematic analysis for the Global Burden of Disease Study 2017. Lancet.

[3] Kang DH, Ha SK (2014). Uric acid puzzle: Dual role as anti-oxidantand pro-oxidant. Electrolyte and Blood Pressure.

[4] Sautin YY, Johnson RJ. Uric acid: The oxidant-antioxidant paradox. In: Nucleosides, Nucleotides and Nucleic Acids. NIH Public Access; 2008. p. 608–19. doi:10.1080/15257770802138558. 10.1080/15257770802138558 PMC2895915 18600514

[5] Barata R, Cardoso F, Pereira T (2020). Hyperuricemia in Chronic Kidney Disease: a role yet to be explained. Port J Nephrol Hypertens.

[6] Johnson RJ, Nakagawa T, Jalal D, Sánchez-Lozada LG, Kang DH, Ritz E (2013). Uric acid and chronic kidney disease: Which is chasing which?. Nephrology Dialysis Transplantation.

[7] Li L, Yang C, Zhao Y, Zeng X, Liu F, Fu P. Is hyperuricemia an independent risk factor for new-onset chronic kidney disease?: A systematic review and meta-analysis based on observational cohort studies. BMC Nephrology. 2014;15. doi:10.1186/1471-2369-15-122. 10.1186/1471-2369-15-122 PMC4132278 25064611

[8] Chonchol M, Shlipak MG, Katz R, Sarnak MJ, Newman AB, Siscovick DS (2007). Relationship of Uric Acid With Progression of Kidney Disease. Am J Kidney Dis.

[9] Gu L, Huang L, Wu H, Lou Q, Bian R (2017). Serum uric acid to creatinine ratio: A predictor of incident chronic kidney disease in type 2 diabetes mellitus patients with preserved kidney function. Diabetes Vasc Dis Res.

[10] Liu WC, Hung CC, Chen SC, Yeh SM, Lin MY, Chiu YW (2012). Association of Hyperuricemia with renal outcomes, cardiovascular disease, and mortality. Clin J Am Soc Nephrol.

[11] Kumagai T, Ota T, Tamura Y, Chang WX, Shibata S, Uchida S (2017). Time to target uric acid to retard CKD progression. Clin Exp Nephrol.

[12] Weiner DE, Tighiouart H, Elsayed EF, Griffith JL, Salem DN, Levey AS (2008). Uric acid and incident kidney disease in the community. J Am Soc Nephrol.

[13] Sengsuk J, Tangvarasittichai O, Tangvarasittichai S (2018). Serum Uric Acid to Creatinine Ratio as a Marker of Estimated Glomerular Filtration Rate in Type 2 Diabetes Patients. Madridge J Diabetes.

[14] Kawamoto R, Ninomiya D, Kikuchi A, Akase T, Kasai Y, Ohtsuka N (2019). Serum uric acid to creatinine ratio is a useful predictor of renal dysfunction among diabetic persons. Diabetes Metab Syndr Clin Res Rev.

[15] Chunlei Y, Liubao G, Tao W, Changying X (2019). The association between serum uric acid to creatinine ratio and renal disease progression in type 2 diabetic patients in Chinese communities. J Diabetes Complications.

[16] Tao J, Shen X, Li J, Cha E, Gu P-P, Liu J (2020). Serum uric acid to creatinine ratio and metabolic syndrome in postmenopausal Chinese women. Medicine (Baltimore).

[17] Al-Daghri NM, Al-Attas OS, Wani K, Sabico S, Alokail MS. Serum Uric Acid to Creatinine Ratio and Risk of Metabolic Syndrome in Saudi Type 2 Diabetic Patients. Sci Rep. 2017;7. doi:10.1038/s41598-017-12085-0. 10.1038/s41598-017-12085-0 PMC5608718 28935934

[18] Zhang H (2019). Study on the Risk of Metabolic Syndrome Based on UA/Cr Analysis. Adv Emerg Med.

[19] Barroso WKS, Rodrigues CIS, Bortolotto LA, Mota-Gomes MA, Brandão AA, Feitosa AD de M, et al. Diretrizes Brasileiras de Hipertensão Arterial-2020. Arq Bras Cardiol. 2021;116:516–658. doi:10.36660/abc.20201238. 10.36660/abc.20201238 PMC9949730 33909761

[20] Matsudo S, Araújo T, Matsudo V, Andrade D, Andrade E, Oliveira LC (2012). Questinário internacional de atividade física (IPAQ): estudo de validade e reprodutibilidade no Brasil. Rev Bras atividade física saúde.

[21] Jelliffe DB (1968). Evaluación del estado de nutrición de la comunidad.

[22] World Health Organization. Obesity: preventing and managing the global epidemic : report of a WHO consultation. World Health Organization; 2000. 11234459

[23] Lipschitz DA. Screening for nutritional status in the elderly. Prim Care. 1994;21:55–67. http://www.ncbi.nlm.nih.gov/pubmed/8197257. Accessed 19 Apr 2018. 8197257

[24] Kidney Disease: Improving Global Outcomes (KDIGO) CKD Work. KDIGO 2012 Clinical Practice Guideline for the Evaluation and Management of Chronic Kidney Disease. Kidney Int Suppl. 2013;3:1–150. http://www.kidney-international.org.

[25] Motta VT, Oliveira Filho PF de. SPSS: análise de dados biomédicos. 2009;:xiv,334-xiv,334.

[26] Obermayr RP, Temml C, Gutjahr G, Knechtelsdorfer M, Oberbauer R, Klauser-Braun R (2008). Elevated uric acid increases the risk for kidney disease. J Am Soc Nephrol.

[27] Hsieh Y-P, Chang C-C, Yang Y, Wen Y-K, Chiu P-F, Lin C-C (2017). The role of uric acid in chronic kidney disease patients. Nephrology.

[28] Son Y Bin, Yang JH, Kim MG, Jo SK, Cho WY, Oh SW. The effect of baseline serum uric acid on chronic kidney disease in normotensive, normoglycemic, and non-obese individuals: A health checkup cohort study. PLoS One. 2021;16 1 January 2021:e0244106. doi:10.1371/journal.pone.0244106. 10.1371/journal.pone.0244106 PMC7840038 33503029

[29] Russo E, Viazzi F, Pontremoli R, Barbagallo CM, Bombelli M, Casiglia E, et al. Association of uric acid with kidney function and albuminuria: the Uric Acid Right for heArt Health (URRAH) Project. J Nephrol. 2021;:1–11. doi:10.1007/s40620-021-00985-4. 10.1007/s40620-021-00985-4 PMC8803667 33755930

[30] Paravicini TM, Touyz RM. NADPH oxidases, reactive oxygen species, and hypertension: clinical implications and therapeutic possibilities. Diabetes care. 2008;31 Supplement 2:S170–80. doi:10.2337/dc08-s247. 10.2337/dc08-s247 18227481

[31] Gliozzi M, Malara N, Muscoli S, Mollace V (2016). The treatment of hyperuricemia. Int J Cardiol.

[32] Kielstein JT, Pontremoli R, Burnier M (2020). Management of Hyperuricemia in Patients with Chronic Kidney Disease: a Focus on Renal Protection. Current Hypertension Reports.

[33] Mulay SR (2019). Multifactorial functions of the inflammasome component NLRP3 in pathogenesis of chronic kidney diseases. Kidney International.

[34] Ephraim RKD, Awuku YA, Numekevor P, Botchway F, Adoba P, Dadzie EK, et al. Serum Uric acid is a better indicator of kidney impairment than serum uric acid to creatine ratio; a cross sectional study of type 2 diabetes mellitus patients. J Diabetes Metab Disord. 2021;:1–8. doi:10.1007/s40200-021-00746-x. 10.1007/s40200-021-00746-x PMC8212335 34178839

[35] Lanaspa MA, Andres-Hernando A, Kuwabara M (2020). Uric acid and hypertension. Hypertension Research.

[36] De Becker B, Borghi C, Burnier M, Van De Borne P (2019). Uric acid and hypertension: A focused review and practical recommendations. Journal of Hypertension.

[37] Grayson PC, Young Kim S, Lavalley M, Choi HK (2011). Hyperuricemia and incident hypertension: A systematic review and meta-analysis. Arthritis Care and Research.

[38] Kuwabara M, Niwa K, Nishi Y, Mizuno A, Asano T, Masuda K (2014). Relationship between serum uric acid levels and hypertension among Japanese individuals not treated for hyperuricemia and hypertension. Hypertens Res.

[39] Ali N, Mahmood S, Islam F, Rahman S, Haque T, Islam S, et al. Relationship between serum uric acid and hypertension: a cross-sectional study in Bangladeshi adults. Sci Rep. 2019;9. doi:10.1038/s41598-019-45680-4. 10.1038/s41598-019-45680-4 PMC6588567 31227765

[40] Bellomo G, Venanzi S, Verdura C, Saronio P, Esposito A, Timio M (2010). Association of uric acid with change in kidney function in healthy normotensive individuals. Am J Kidney Dis.

[41] Sedaghat S, Hoorn EJ, Van Rooij FJA, Hofman A, Franco OH, Witteman JCM, et al. Serum uric acid and chronic kidney disease: The role of hypertension. PLoS One. 2013;8. doi:10.1371/journal.pone.0076827. 10.1371/journal.pone.0076827 PMC3827035 24265674

